# Assessment of social media use as an educational intervention on antimicrobial resistance targeted at nurses

**DOI:** 10.1590/1980-220X-REEUSP-2025-0388en

**Published:** 2026-07-27

**Authors:** Lígia Maria Abraão, Chayenne Mika Matsumoto Tonheiro, Tamires Moraes Leite da Silva, Viviane Cristina de Lima Gusmão, Caroline Lopes Ciofi-Silva, Eric Gustavo Almeida, Adriana Maria da Silva Felix, Letícia F. Britto-Costa, Tatiane Garcia do Carmo Flausino, Rosely Moralez de Figueiredo, Maria Clara Padoveze

**Affiliations:** 1 Universidade de São Paulo, Escola de Enfermagem, São Paulo, SP, Brazil.; 2 Hospital Estadual Vila Alpina, São Paulo, SP, Brazil.; 3 Hospital Santa Helena, Santo André, SP, Brazil.; 4 Universidade Estadual de Campinas, Faculdade de Enfermagem, Campinas, SP, Brazil.; 5 Hospital Universitário Clementino Fraga Filho, Rio de Janeiro, RJ, Brazil.; 6 Faculdade Sírio-Libanês, São Paulo, SP, Brazil.; 7 Universidade Federal de São Carlos, São Carlos, SP, Brazil.

**Keywords:** Nursing, Drug Resistance, Microbial, Antimicrobial Stewardship, Social Networking, Education, Nursing

## Abstract

**Objective:**

To assess social media user engagement with educational strategies aimed at raising nurses’ awareness of antimicrobial resistance (AMR) and the Antimicrobial Stewardship Program (ASP).

**Method:**

A prospective study conducted between October 2023 and October 2024 using data from the Brazilian Nurses Network Tackling Antimicrobial Resistance (REBRAN) on Instagram^®^. Engagement was measured biweekly using platform metrics, including followers, impressions, views, reach, and interactions. The correlation between REBRAN membership, follower growth, and the highest-engagement content was analyzed using descriptive statistics and Pearson’s correlation coefficient.

**Results:**

The number of members increased from 214 to 436 (136%), and the number of followers increased from 507 to 1,500, with a strong positive correlation (r = 0.97). Playful content, such as analogies with movies and commemorative dates, generated greater engagement than technical content. The best performance occurred in June 2024, with 15,000 impressions, 209 views, and a reach of 7,400 accounts.

**Conclusion:**

Playful and interactive content on social media promotes user engagement regarding AMR, increasing awareness and encouraging evidence-based practices in ASPs.

## INTRODUCTION

Antimicrobial resistance (AMR) in humans and animals represents one of the ten major global public health threats worldwide^(1)^. The rational use of antimicrobials, particularly through Antimicrobial Stewardship Programs (ASPs), is recognized as essential for preventing the emergence and spread of resistant organisms^(1,2,3)^.

Traditionally, ASPs have been led by infectious disease physicians and pharmacists. However, professional organizations and experts in the field increasingly advocate for a collaborative and interdisciplinary approach that includes nurses^(4,5,6)^, considering the multiple activities these professionals perform in healthcare institutions that contribute to combating AMR^(4,7,8,9,10)^. Despite nurses’ involvement in ASP-related activities, there remains a notable lack of awareness and knowledge regarding antimicrobial stewardship among Brazilian nurses^(11,12)^.

To strengthen, support, and facilitate nurses’ participation and influence in ASPs, thereby positively impacting AMR control efforts, the Brazilian Nurses Network Tackling Antimicrobial Resistance (REBRAN) was established. REBRAN is a technical cooperation network primarily composed of nurses, aiming to promote scientific discussions, develop research initiatives, and contribute to knowledge dissemination and professional engagement concerning AMR-related challenges in Brazil. Nurses interested in this topic may voluntarily join REBRAN. One of the network’s communication channels includes a professional Instagram^®^ account designed to disseminate content related to AMR and ASPs^(13)^.

Social media platforms have become powerful communication tools due to their broad reach, allowing rapid dissemination of information, interaction with specific audiences, and engagement of healthcare professionals^(14,15)^. Platforms such as Instagram^®^ facilitate not only content sharing but also the creation of interactive and engaging experiences that may enhance communication and contribute to increasing awareness of issues such as AMR^(16,17)^.

Social media performance can be assessed using informational metrics including visibility (or views), influence, participation, and engagement^(15)^. *Views* are defined as indicators of the frequency with which content is displayed^(18)^. *Influence* refers to the ability to affect attitudes and behaviors^(19)^. *Participation* encompasses active responses such as commenting, sharing, and replying^(20)^. User *engagement* occurs when individuals actively interact with content on a social network by performing actions such as clicking, commenting, or liking—commonly referred to as “interactions”. These interactions are measured relative to publication reach or number of followers, resulting in the engagement rate^(21)^. Generally, this indicator is calculated based on the “3 Cs” triad: comments, clicks, and content sharing^(15)^.

Historically, the use of social media in health research was initially met with skepticism within the scientific community. However, the number of publications on this topic in scientific journals has increased considerably in recent years, reflecting recognition of the potential applications of these strategies^(22,23,24)^. Social media applications in health research are broad and diverse and already include interventions designed to increase awareness, including awareness regarding AMR^(25)^. This transformation in global communication has important implications for public health and nursing practice, as social media platforms can contribute to enhancing nurses’ visibility in healthcare delivery. Within this context, the aim of this study was to assess user engagement on a social media platform through educational strategies designed to increase nurses’ awareness regarding AMR and ASPs.

## METHODS

### Study Design

This was a prospective observational study assessing user engagement on a social media platform following the implementation of an educational strategy.

### Setting and Population

The study was conducted in Brazil between October 2023 and October 2024. The target population consisted of nurses affiliated with REBRAN and followers of the network’s Instagram^®^ account: “@rebran2022”. However, because Instagram^®^ is an open-access social media platform, outcome indicators included any individuals or organizations that interacted with the REBRAN Instagram^®^ profile during the study period, not exclusively nurses.

### Social Media Educational Strategy

**Figure 1:**
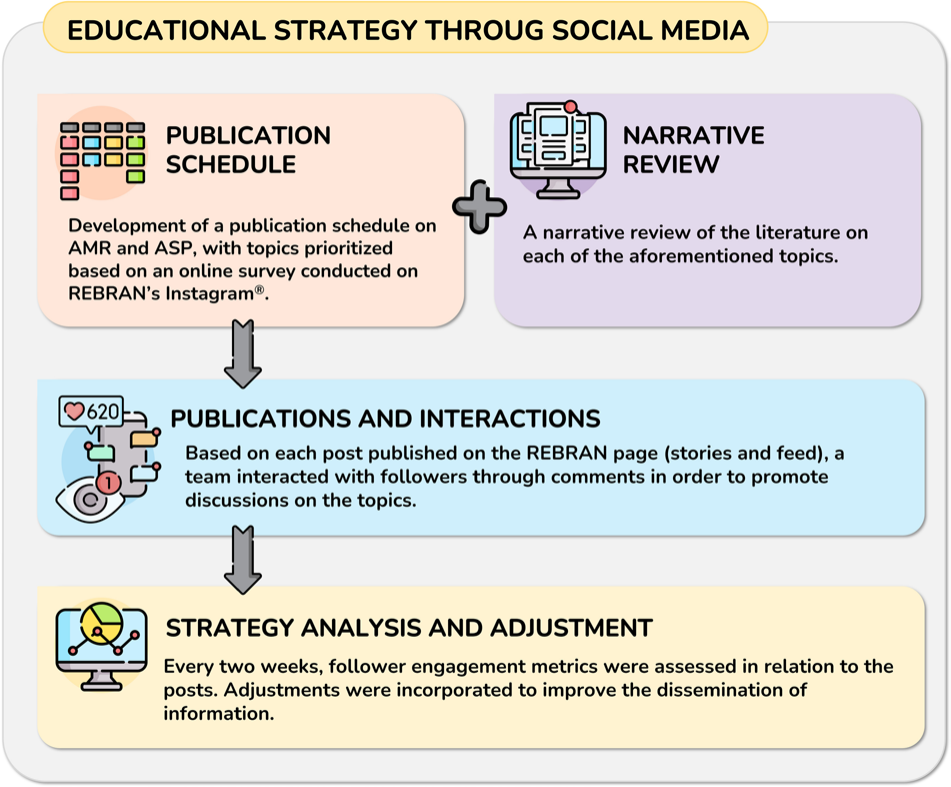
Schematic representation of the social media educational strategy designed to promote nurses’ engagement with antimicrobial resistance and antimicrobial stewardship program content – São Paulo, SP, Brazil, 2025.

**Source:** Authors, 2025.

The educational strategy was implemented through Instagram^®^, currently the most widely used social media platform in Brazil^(26)^. The intervention implementation strategy was organized into three stages (Figure 1). All activities were developed by a multidisciplinary team of researchers from academic institutions and healthcare organizations and coordinated by the REBRAN leadership team.

### Data Collection

To collect Instagram^®^-related metrics, a specialized social media monitoring platform, V-tracker^®^, was used. This platform integrates social listening tools with artificial intelligence-generated insights. Additional information, including platform insights and professional dashboard indicators from Instagram^®^ (views, interactions, and total number of followers), was extracted directly from the restricted-access professional dashboard on a biweekly basis by designated members of the REBRAN social media working group (L.M.A.; C.M.M.T.; and T.M.L.S.) between October 2023 and October 2024.

### Data Analysis and Processing

**Figure 2:**
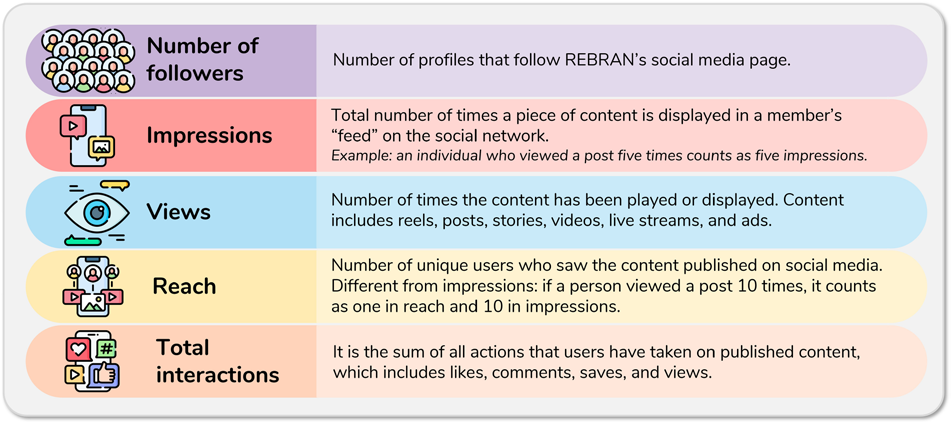
Social media monitoring metrics used in the study – São Paulo, SP, Brazil, 2025.

**Source:** Authors, 2025.

Following data collection, the information was transferred to a Microsoft Excel^®^ spreadsheet for statistical analysis. Specific social media monitoring metrics were applied to assess performance indicators (Figure 2).

Data were analyzed according to temporal trends throughout the selected study period (12 months). To assess the relationship between the number of followers on REBRAN’s Instagram^®^ page and the number of officially registered REBRAN members, Pearson’s correlation analysis was performed. This statistical method quantifies the strength and direction of the linear association between two continuous quantitative variable^(27)^. Pearson’s correlation coefficient (r) ranges from −1 to +1, with values closer to +1 indicating a strong positive linear correlation^(28)^. Finally, the study sought to identify the content themes associated with the highest levels of user engagement on REBRAN’s Instagram^®^ page.

### Ethical Considerations

This study was approved by the Research Ethics Committee of the School of Nursing at *Universidade de São Paulo*, under Protocol number 5,953,109 (2024).

## RESULTS

At the beginning of the implementation of the social media educational strategy, REBRAN had 214 officially registered members. By the end of the study period, this number had increased to 436 members, representing a growth of 136%. A similar trend was observed on the Instagram^®^ profile, where the number of followers increased from 507 to 1,500, showing continuous growth throughout the study period.

**Figure 3:**
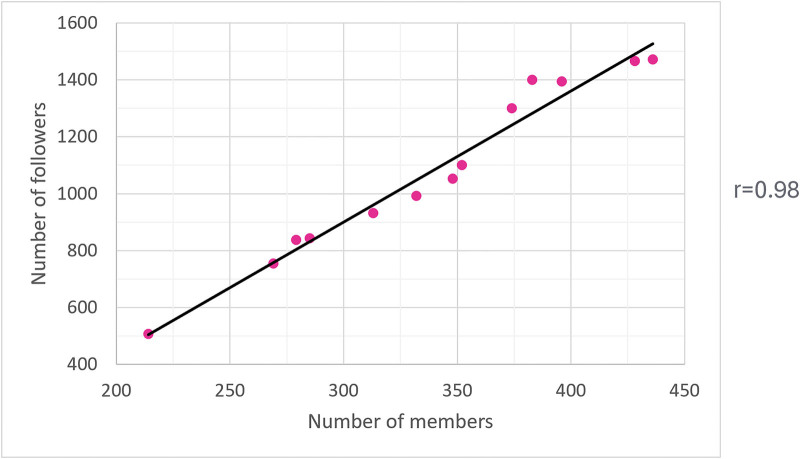
Correlation analysis between the number of affiliated members and the number of followers on Brazilian Nurses Network Tackling Antimicrobial Resistance’s Instagram^®^ profile – São Paulo, SP, Brazil, 2025.

Correlation analysis between the number of affiliated members and Instagram^®^ followers demonstrated a very strong positive association (r = 0.98), highlighting the potential of social media as a tool to expand the network’s reach (Figure 3).

The main topics of interest were published as post-format content using the trend strategy (a term that refers to content that reaches a peak of popularity on social media platforms for a certain period of time). The topics addressed included the structure of REBRAN, AMR, healthcare-associated infections (HAIs), antimicrobials, the role of nurses in ASPs, blood cultures, the trend inspired by the movie “Inside Out 2”^(29)^, which related emotions to AMR control, the Halloween trend, which presented an analogy about AMR, myths and facts about AMR, and the alert issued by the Brazilian Health Regulatory Agency regarding vancomycin-resistant Staphylococcus aureus.

Regarding the social media metrics assessed throughout the study period, substantial growth was observed. In October 2023, publications achieved an average of 1,600 impressions, 52.2 views, and reached 701 accounts. By October 2024, these metrics had increased to 5,400 impressions, 80.7 views, and a reach of 3,100 accounts.

Among all published content, the most notable performance occurred in June 2024, when posts relating ideas and characters from the animated movie “Inside Out 2” (produced by Walt Disney Pictures and Pixar Animation Studios) to AMR control achieved the highest engagement indicators, including impressions, views, and account reach (15,000, 209, and 7,400, respectively). In November 2023, REBRAN initiatives during the World Antimicrobial Resistance Awareness Week, which included live sessions, discussions, and educational materials, also demonstrated substantial performance (4,800 impressions, 161 views, and a reach of 1,700 accounts). Similarly, in October 2024, Halloween-themed publications employing playful approaches related to AMR achieved high reach and engagement metrics (4,600 impressions, 54 views, and reach of 3,300 accounts).

Conversely, the lowest performance in terms of total interactions and reach was observed in January 2024, when content related to HAIs—specifically the epidemiological chain—was published.

## DISCUSSION

Social media platforms are currently considered among the primary tools for promoting engagement in contemporary society. However, their full potential remains underutilized. A systematic review published in 2022 investigated the effectiveness of interventions aimed at increasing public awareness regarding AMR and identifying which interventions most strongly influenced public behavior. Among the eligible studies included in the review, none employed social media resources as part of their intervention strategies^(30)^.

The use of social media to raise awareness, engage audiences, and disseminate information to healthcare professionals and healthcare users represents a powerful tool that could become a cornerstone in the global fight against AMR^(31)^, given its broad reach, low cost, and substantial dissemination capacity. This strategy may be particularly relevant in low- and middle-income countries, where access to health literacy resources does not always reach all segments of the population^(31)^.

The transformation driven by social media has enabled rapid health communication through strategic and targeted tools capable of reaching specific audiences with high penetration and relatively low cost. However, a balance must be achieved between the benefits of these strategies and the potential harms associated with online misinformation^(32)^.

Currently, social media represents an ambivalent environment for healthcare professionals regarding information dissemination. While social media platforms facilitate the rapid dissemination of information beyond geographical boundaries, influence attitudes, and increase users’ awareness, they can also be used to spread fake news and misinformation^(33)^. A review demonstrated that misinformation and false news have a higher likelihood of spreading on social media than evidence-based scientific communication posts. Furthermore, such misinformation may promote inaccurate beliefs and compromise future understanding, particularly when compared with accurate content disseminated through educational profiles^(34)^.

Considering this scenario, healthcare professionals should increasingly engage with social media dissemination mechanisms to promote scientifically grounded information and support evidence-based practices in healthcare settings. By adhering to ethical and legal standards governing professional practice, social media engagement can contribute to professional and societal recognition while demonstrating commitment to scientific dissemination and its application in healthcare^(35)^. Therefore, the deliberate and strategic use of social media to disseminate accurate health information—including content specifically directed toward healthcare professionals, as in the present study—should be considered part of broader AMR awareness initiatives.

In the present study, posts with playful and entertaining characteristics generated superior engagement metrics, suggesting that this strategy may represent a promising pathway for future AMR awareness campaigns. This observation is supported by international experiences, such as the Spanish SWICEU project (a consortium involving more than 30 universities from Spain and Portugal, sponsored by the Spanish Society of Microbiology), which used infographics, videos, and pop-culture-inspired gamification strategies to increase awareness regarding AMR among young people and demonstrated substantial engagement and dissemination outcomes^(16)^.

Another relevant aspect concerns the role of social media in facilitating interactions among healthcare professionals. Studies indicate that platforms such as Twitter^®^ (currently X^®^) and Facebook^®^ expand real-time scientific communication, encourage experience sharing, and contribute to the consolidation of best practices in infection prevention and ASPs. A study conducted by Pisano et al.^(36)^ demonstrated that educational social media content not only increased medical residents’ knowledge regarding antimicrobials but also encouraged the use of protocol-based clinical resources, highlighting the educational potential of these tools.

Additionally, it is important to consider that REBRAN members consist predominantly of nurses directly involved in HAI prevention and control activities, which may have influenced the lower engagement observed for posts addressing theoretical concepts. One possible explanation is that these professionals perceived themselves as already having substantial familiarity with these topics or considered the format insufficiently innovative or attractive to encourage interaction. Therefore, more creative and accessible content formats may have greater impact, particularly for strengthening engagement with AMR-related topics^(14)^.

Finally, Instagram^®^, due to its more informal and dynamic nature, facilitates experience exchange, supports collaborative learning processes, and offers diverse resources for content development^(37)^. These characteristics enhance its potential as a teaching-learning and AMR awareness tool and may reinforce its strategic value for healthcare professionals and the general population.

This study has several limitations. First, it was not possible to identify which REBRAN members demonstrated greater interaction with the social media platform, preventing a more detailed analysis of engagement profiles across activities. Additionally, because the social media platform was open access, it was not possible to determine whether interactions were performed exclusively by nurses. Finally, the study period was limited to one year, preventing conclusions regarding the long-term sustainability of this awareness strategy.

## CONCLUSION

The findings of this study demonstrate that social media platforms, particularly Instagram^®^, may serve as strategic tools for promoting AMR awareness. Analysis of engagement metrics revealed that playful and interactive content generated higher engagement levels, suggesting that the use of creative communication approaches may enhance the impact of digital health campaigns.

In this context, initiatives such as those developed by REBRAN demonstrate that scientific communication can be innovative, engaging, and effective, representing a model with potential for expansion and replication across broader public health contexts.

## DATA AVAILABILITY

The entire dataset supporting the results of this study is available upon request to the corresponding author.
