# Supplementary material for: Assessment of social media use as an educational intervention on antimicrobial resistance targeted at nurses
Source: Rev Esc Enferm USP. 2026 Jul 27;60:e20250388. doi: 10.1590/1980-220X-REEUSP-2025-0388en (PMC13411132; doi:10.1590/1980-220X-REEUSP-2025-0388en)
Supplement: Translation

# Translation: Avaliação do uso de rede social como intervenção educativa sobre resistência antimicrobiana direcionada a enfermeiros

**DOI:** 10.1590/1980-220X-REEUSP-2025-0388pt

**Authors:** Lígia Maria Abraão, Chayenne Mika Matsumoto Tonheiro, Tamires Moraes Leite da Silva, Viviane Cristina de Lima Gusmão, Caroline Lopes Ciofi-Silva, Eric Gustavo Almeida, Adriana Maria da Silva Felix, Letícia F. Britto-Costa, Tatiane Garcia do Carmo Flausino, Rosely Moralez de Figueiredo, Maria Clara Padoveze

**Affiliations:** 1 Universidade de São Paulo, Escola de Enfermagem, São Paulo, SP, Brasil.; 2 Hospital Estadual Vila Alpina, São Paulo, SP, Brasil.; 3 Hospital Santa Helena, Santo André, SP, Brasil.; 4 Universidade Estadual de Campinas, Faculdade de Enfermagem, Campinas, SP, Brasil.; 5 Hospital Universitário Clementino Fraga Filho, Rio de Janeiro, RJ, Brasil.; 6 Faculdade Sírio-Libanês, São Paulo, SP, Brasil.; 7 Universidade Federal de São Carlos, São Carlos, SP, Brasil.

**Keywords:** Enfermagem, Resistência a Medicamentos, Gestão de Antimicrobianos, Redes Sociais, Educação em Enfermagem

## Abstract

**Objetivo:**

Avaliar o engajamento de usuários de uma rede social mediante estratégias educativas direcionadas à conscientização dos enfermeiros sobre resistência antimicrobiana (RAM) e o Programa de Gerenciamento de Antimicrobianos (PGA).

**Método:**

Estudo prospectivo, realizado entre outubro de 2023 e outubro de 2024, com dados da Rede Brasileira de Enfermeiros para Enfrentamento da Resistência Antimicrobiana (REBRAN) no *Instagram* ^®^. O engajamento foi medido quinzenalmente por métricas da plataforma, como seguidores, impressões, visualizações, alcance e interações. Analisou-se a correlação entre membros da REBRAN, seguidores e conteúdos de maior engajamento por meio de estatística descritiva e do coeficiente de Pearson.

**Resultados:**

O número de membros aumentou de 214 para 436 (136%), e os seguidores, de 507 para 1.500, com forte correlação positiva (r = 0,97). Conteúdos lúdicos, como analogias com filmes e datas comemorativas, tiveram maior engajamento que conteúdos técnicos. O melhor desempenho ocorreu em junho de 2024, com 15.000 impressões, 209 visualizações e alcance de 7.400 contas.

**Conclusão:**

Conteúdos lúdicos e interativos em redes sociais favorecem o engajamento de usuários sobre a RAM, ampliando a conscientização e incentivando práticas baseadas em evidências nos PGAs.

## INTRODUÇÃO

A resistência antimicrobiana (RAM) em humanos e animais representa uma das dez principais ameaças globais em saúde^(1)^. O uso racional de antimicrobianos, capitaneado especialmente pelos Programas de Gestão de Antimicrobianos (PGAs), é reconhecido como essencial na prevenção do desenvolvimento de organismos resistentes^(1,2,3)^.

Os PGAs tradicionalmente têm sido conduzidos por infectologistas e farmacêuticos. No entanto, organizações de classe e especialistas na área recomendam uma abordagem colaborativa e interdisciplinar, com a inclusão de enfermeiros^(4,5,6)^, considerando que esses profissionais desempenham diversas atividades pertinentes ao combate da RAM nas instituições de saúde^(4,7,8,9,10)^. Apesar das ações dos enfermeiros conectadas aos PGAs, ainda existe uma notável falta de conhecimento e conscientização desses profissionais brasileiros sobre a gestão de antimicrobianos^(11,12)^.

Com a proposta de aproximar, subsidiar e fortalecer enfermeiros para atuar e influenciar os PGAs, impactando positivamente o controle da RAM, foi criada a Rede Brasileira de Enfermeiros para Enfrentamento da Resistência Antimicrobiana (REBRAN). Trata-se de um grupo de cooperação técnica formado, prioritariamente, por enfermeiros, com o objetivo de fomentar discussões científicas, desenvolver pesquisas e contribuir para a difusão de conhecimento e de engajamento dos enfermeiros diante da problemática da RAM no Brasil. Os enfermeiros interessados sobre o tema podem se filiar à REBRAN. Esse grupo de cooperação possui como um dos seus canais de comunicação uma conta do tipo “profissional” na rede social *Instagram* ^®^ para difusão de conteúdos relacionados à RAM e ao PGA^(13)^.

As redes sociais têm se consolidado como uma ferramenta de comunicação de grande alcance, possibilitando a rápida difusão de informações, a interação com públicos específicos e o engajamento de profissionais de saúde^(14,15)^. Plataformas como o *Instagram* ^®^ possibilitam não apenas o compartilhamento de conteúdo, mas também a criação de experiências interativas e lúdicas, que podem favorecer a comunicação, contribuindo para o fortalecimento da conscientização sobre temas como a RAM^(16,17)^.

O desempenho de uma rede social pode ser mensurado por métricas informacionais, como visibilidade (ou visualizações), influência, participação e engajamento^(15)^. As *visualizações* são definidas como indicadores de frequência com os quais os conteúdos são exibidos^(18)^. A *influência* é a capacidade de impactar atitudes e comportamentos^(19)^. Já a *participação* refere-se ao ato de comentar, compartilhar e responder de forma ativa^(20)^. Por fim, o *engajamento* do usuário ocorre quando ele realiza uma ação ativa em relação ao conteúdo em uma rede social, como clicar, comentar ou curtir — ações conhecidas como “interações”. Essas interações são mensuradas em relação ao alcance da publicação ou ao número de seguidores, resultando na chamada “taxa de engajamento”^(21)^. Em geral, esse indicador é calculado a partir da soma da tríade dos “3 Cs”: curtidas, comentários e compartilhamento^(15)^.

Historicamente, a utilização das mídias sociais na área de pesquisa em saúde foi recebida com certo ceticismo por parte da comunidade científica. Porém, atualmente, tem crescido o número de publicações sobre o tema em revistas científicas, considerando as potencialidades dessa estratégia^(22,23,24)^. As aplicações das mídias sociais na pesquisa em saúde são amplas e variadas, e já incluem estratégias para aumentar a conscientização, inclusive sobre a RAM^(25)^. Essa transformação na comunicação global tem implicações significativas para a saúde pública e para a enfermagem, uma vez que as redes sociais podem ser utilizadas para maior visibilidade do enfermeiro nas práticas de cuidado à saúde. Diante desse contexto, o objetivo deste estudo foi avaliar o engajamento de usuários de uma rede social mediante estratégias educativas direcionadas para conscientização dos enfermeiros sobre RAM e PGA.

## MÉTODO

### Desenho do Estudo

Trata-se de um estudo observacional prospectivo que avaliou o engajamento de usuários de uma rede social por meio da implementação de uma estratégia educativa.

### Local e População

O estudo foi realizado no Brasil no período compreendido entre outubro de 2023 e outubro de 2024. A população-alvo foi composta por enfermeiros afiliados à REBRAN e seguidores da rede no *Instagram* ^®^: “@rebran2022”. No entanto, por se tratar de uma rede social de comunicação aberta, os indicadores de resultados incluíram qualquer indivíduo/organização que interagiu com o perfil da REBRAN no *Instagram* ^®^ no período proposto, não apenas enfermeiros.

### Estratégia Educativa por Rede Social

A estratégia educativa foi implementada no *Instagram* ^®^, principal rede social em uso no Brasil^(26)^. A estratégia para implementação da intervenção foi organizada em três etapas (Figura 1). Todas as ações foram desenvolvidas por uma equipe de pesquisadores procedentes de diferentes instituições acadêmicas e serviços de saúde, lideradas pela equipe de coordenação da REBRAN.

**Figura 1:** Figura esquemática da estratégia educativa por rede social para engajamento de enfermeiros nos conteúdos de resistência antimicrobiana e Programa de Gestão de Antimicrobianos – São Paulo, SP, Brasil, 2025.

**Fonte:** Autores, 2025.

### Coleta de Dados

Para coleta dos dados relacionados às métricas do *Instagram* ^®^, foi utilizada uma plataforma específica para monitoramento de redes sociais, a *V-tracker* ^®^, que utiliza ferramentas que unem *Social Listening* e *insights* elaborados por Inteligência Artificial. As informações complementares, como *insights* ou indicadores do painel profissional do *Instagram* ^®^ (visualizações, interações e total de seguidores), foram extraídas diretamente da área restrita quinzenalmente, por membros designados do grupo de trabalho das redes sociais da REBRAN (L.M.A.; C.M.M.T. e T.M.L.S.), entre outubro de 2023 e outubro de 2024.

### Análise e Tratamento dos Dados

Após a coleta, os dados foram transferidos para uma planilha do programa *Microsoft Excel* ^®^ para análise estatística, no qual foram utilizadas métricas específicas de monitoramento das redes sociais (Figura 2).

**Figura 2:** Métricas utilizadas para monitoramento em redes sociais. São Paulo – SP, Brasil, 2025.

**Fonte:** Autores, 2025.

As informações foram analisadas em relação à tendência temporal ao longo do período selecionado (12 meses). Para avaliar a relação entre as variáveis, o número de seguidores na página da REBRAN no *Instagram* ^®^ e o número de membros oficialmente registrados na REBRAN, foi aplicada a correlação de Pearson, que quantifica a força e a direção da associação linear entre duas variáveis quantitativas contínuas^(27)^. O coeficiente de correlação de Pearson (r) varia entre -1 e 1, em que valores próximos de 1 indicam forte correlação direta (positiva)^(28)^. Por fim, buscou-se identificar os temas publicados de maior engajamento entre os usuários da página da REBRAN no *Instagram* ^®^.

### Aspectos Éticos

Esta pesquisa foi aprovada pelo Comitê de Ética em Pesquisa da Escola de Enfermagem da Universidade de São Paulo, sob Parecer nº 5.953.109 de 2024.

## RESULTADOS

No início da implementação estratégia educativa por rede social, a REBRAN contava com 214 membros oficialmente registrados, número que aumentou para 436 ao final do período, representando crescimento de 136%. Tendência semelhante foi observada no perfil do *Instagram* ^®^, cujo número de seguidores passou de 507 para 1.500, com incremento contínuo ao longo do período avaliado. A análise de correlação entre membros e seguidores revelou correlação muito forte (r = 0,98), demonstrando o potencial da rede social como ferramenta para expandir o alcance da rede (Figura 3).

**Figura 3:** Análise de correlação entre o número de membros afiliados e o número de seguidores no Instagram^®^ da Rede Brasileira de Enfermeiros para Enfrentamento da Resistência Antimicrobiana – São Paulo, SP, Brasil, 2025.

Os principais temas de interesse foram publicados em conteúdos em formato de *post* utilizando a estratégia de *trends* (termo que significa “tendência” e dá nome aos conteúdos que atingem um pico de popularidade nas redes sociais por certo tempo). Os temas abordados incluíram a estrutura da REBRAN, a RAM, as infecções relacionadas à assistência à saúde (IRAS), os antimicrobianos, o papel do enfermeiro nos PGAs, as hemoculturas, a tendência do filme “Divertida Mente 2”^(29)^, que relaciona as emoções com o controle da RAM, a tendência de *Halloween*, que apresenta uma analogia sobre a RAM, os mitos e verdades sobre a RAM, e o alerta da Agência Nacional de Vigilância Sanitária sobre *Staphylococcus aureus* resistente à vancomicina.

Em relação às métricas da rede social avaliadas ao longo do período, observou-se um crescimento expressivo: as publicações, que em outubro de 2023 registravam em média 1.600 impressões, 52,2 visualizações e alcance de 701 contas, passaram em outubro de 2024 para 5.400 impressões, 80,7 visualizações e alcance de 3.100 contas.

Entre os conteúdos publicados, o destaque ocorreu em junho de 2024, quando a estratégia de *posts* relacionando ideias e personagens do filme de animação “Divertida Mente 2” (produzido pelos estúdios *Walt Disney, Pictures, Pixar Animation Studios*) ao controle da RAM alcançou os melhores indicadores de engajamento, com maior número de impressões, visualizações e alcance (15.000, 209 e 7.400, respectivamente). Em novembro de 2023, as ações da REBRAN para a Semana Mundial de Conscientização sobre Resistência Antimicrobiana, que envolveram *lives*, discussões e materiais educativos, também apresentaram resultados relevantes (4.800 impressões, 161 visualizações e alcance de 1.700 contas). Já em outubro de 2024, as publicações alusivas ao *Halloween*, com abordagem lúdica sobre a RAM, obtiveram desempenho expressivo em alcance e engajamento (4.600 impressões, 54 visualizações e alcance de 3.300 contas).

Em contrapartida, o desempenho mais baixo em termos de interações totais e alcance foi observado em janeiro de 2024, quando foi publicado sobre IRAS, especificamente sobre a cadeia epidemiológica (impressões, visualizações e alcance).

## DISCUSSÃO

As redes sociais são consideradas uma das principais ferramentas para gerar engajamento na sociedade atual. Contudo, elas não são exploradas em todo o seu potencial. Estudo de revisão sistemática publicado em 2022 investigou a eficácia das intervenções para melhorar a conscientização do público acerca da RAM, bem como quais intervenções influenciam mais o comportamento público. Esta revisão não encontrou, entre os estudos elegíveis, nenhum que tivesse utilizado recursos das redes sociais em suas estratégias de intervenção^(30)^.

O uso das redes sociais para sensibilizar, engajar e informar profissionais e até mesmo usuários dos sistemas de saúde é uma poderosa ferramenta que poderia se tornar um pilar na luta mundial contra a RAM^(31)^, visto ter um grande alcance, baixo custo e elevada capacidade de alcance. Essa estratégia pode ser particularmente importante em países de baixa e média renda, onde o acesso ao letramento em saúde nem sempre alcança toda a população^(31)^.

A transformação que as redes sociais têm desencadeado possibilita uma rápida comunicação em saúde, com o desenvolvimento de ferramentas estratégicas e direcionadas para públicos específicos com alta capilaridade e baixo custo. Contudo, é necessário encontrar um equilíbrio entre os benefícios dessas estratégias e os danos potenciais relacionados à desinformação *online* ^(32)^.

As redes sociais são, atualmente, um espaço ambivalente no que se refere ao compartilhamento de informações para profissionais de saúde. Se, por um lado, auxiliam na rápida disseminação de informações para além das fronteiras geográficas, influenciam atitudes e aumentam a conscientização dos usuários, por outro, podem ser utilizadas para a propagação de notícias falsas e desinformação^(33)^. Revisão apontou que notícias falsas e desinformação apresentam uma chance maior de se espalhar nas redes sociais em comparação a postagens de divulgação científica baseadas em evidências. Além disso, essas notícias falsas podem favorecer a assimilação de concepções imprecisas, comprometendo a compreensão futura, especialmente quando comparadas a conteúdos acurados divulgados por perfis educativos^(34)^.

Tendo isso em vista, é necessário que profissionais de saúde se apropriem dos mecanismos de alcance das redes sociais para propagar informações embasadas cientificamente e promover as melhores práticas baseadas em evidências nos serviços de saúde. Ao seguir os limites éticos e legais que regem a prática profissional, é possível conquistar reconhecimento social e profissional, demonstrando compromisso com a divulgação científica e sua aplicação no cuidado em saúde^(35)^. Assim sendo, o uso deliberado e planejado da rede social para aumentar a informação correta em saúde, incluindo informações para profissionais, como no caso do presente estudo, deve ser considerado como parte de amplas estratégias de conscientização sobre RAM.

Neste estudo, observou-se que postagens com caráter lúdico e divertido resultaram em melhores métricas de engajamento, sugerindo que esse pode ser um caminho promissor para publicações futuras que visem à conscientização sobre RAM. Essa observação é corroborada por experiências internacionais, como o projeto espanhol SWICEU (grupo de mais de 30 universidades entre Espanha e Portugal, patrocinado pela Sociedade Espanhola de Microbiologia), que utilizou infográficos, vídeos e gamificação inspirados em cultura pop para conscientizar jovens sobre RAM, com resultados expressivos em engajamento e alcance^(16)^.

Outro aspecto relevante é o papel das redes sociais na interação entre profissionais. Estudos apontam que plataformas como *Twitter* ^®^ (atual X^®^) e *Facebook* ^®^ ampliam a comunicação científica em tempo real, estimulando a troca de experiências e a consolidação de boas práticas em controle de infecções e PGAs. Em estudo conduzido por Pisano et al.^(36)^, verificou-se que conteúdos educativos em redes sociais não apenas aumentaram o conhecimento de residentes médicos sobre antimicrobianos, mas também estimularam a utilização de recursos clínicos baseados em protocolos, destacando o potencial pedagógico dessas ferramentas.

Além disso, destaca-se que o perfil de membros da REBRAN, composto majoritariamente por enfermeiros que atuam diretamente na prevenção e no controle de IRAS, pode ter influenciado o menor número de acessos a postagens que abordaram aspectos teóricos de alguns temas. Uma das hipóteses sobre esse resultado é que esses enfermeiros considerem que já têm pleno domínio nesses temas ou que a forma como foram apresentados não foi considerada inovadora ou atraente o suficiente para gerar interação. Assim, conteúdos mais criativos e acessíveis podem ter maior impacto, especialmente no fortalecimento do engajamento com a temática^(14)^.

Por fim, cabe ressaltar que o *Instagram* ^®^, por ser uma rede social de caráter mais informal e dinâmico, favorece a troca de experiências, fomenta um processo de ensino colaborativo e disponibiliza recursos variados para o desenvolvimento de conteúdos^(37)^. Essa característica amplia seu potencial como ferramenta de ensino-aprendizagem e de conscientização sobre RAM, o que pode reforçar seu papel estratégico para profissionais de saúde e para a população em geral.

Este estudo possui limitações, como a dificuldade de identificar entre os membros da REBRAN os que foram mais interativos na rede social, o que não permite detalhar características dos perfis relativos ao engajamento nas diferentes atividades. Devido ao fato de a rede social ser aberta, houve a limitação de não reconhecer se as interações foram feitas exclusivamente por enfermeiros. Por fim, o período do estudo restringiu-se a um ano, o que não permite extrapolar conclusões quanto à sustentabilidade de longo prazo dessa estratégia de conscientização.

## CONCLUSÃO

Os achados deste estudo demonstram que as redes sociais, em especial o Instagram^®^, podem configurar-se como ferramentas estratégicas para a promoção da conscientização sobre RAM. A análise das métricas revelou que postagens lúdicas alcançam maior engajamento, sugerindo que a adoção de linguagens criativas pode potencializar o impacto das campanhas digitais em saúde.

Nesse sentido, iniciativas como a desenvolvida pela REBRAN demonstram que a comunicação científica pode ser inovadora, atrativa e efetiva, representando um modelo que pode ser expandido e replicado em outros contextos de saúde pública.

## DISPONIBILIDADE DE DADOS

Todo o conjunto de dados que dá suporte aos resultados deste estudo está disponível mediante solicitação ao autor correspondente.

# Translation: Evaluación del uso de las redes sociales como intervención educativa sobre la resistencia a los antimicrobianos dirigida a enfermeros

**DOI:** 10.1590/1980-220X-REEUSP-2025-0388es

**Authors:** Lígia Maria Abraão, Chayenne Mika Matsumoto Tonheiro, Tamires Moraes Leite da Silva, Viviane Cristina de Lima Gusmão, Caroline Lopes Ciofi-Silva, Eric Gustavo Almeida, Adriana Maria da Silva Felix, Letícia F. Britto-Costa, Tatiane Garcia do Carmo Flausino, Rosely Moralez de Figueiredo, Maria Clara Padoveze

**Affiliations:** 1 Universidade de São Paulo, Escola de Enfermagem, São Paulo, SP, Brasil.; 2 Hospital Estadual Vila Alpina, São Paulo, SP, Brasil.; 3 Hospital Santa Helena, Santo André, SP, Brasil.; 4 Universidade Estadual de Campinas, Faculdade de Enfermagem, Campinas, SP, Brasil.; 5 Hospital Universitário Clementino Fraga Filho, Rio de Janeiro, RJ, Brasil.; 6 Faculdade Sírio-Libanês, São Paulo, SP, Brasil.; 7 Universidade Federal de São Carlos, São Carlos, SP, Brasil.

**Keywords:** Enfermería, Resistencia a Medicamentos, Programas de Optimización del Uso de los Antimicrobianos, Red Social, Educación en Enfermería

## Abstract

**Objetivo:**

Evaluar la participación de los usuarios de una red social mediante estrategias educativas destinadas a sensibilizar al personal de enfermería sobre la resistencia a los antimicrobianos (RAM) y el Programa de Gestión de Antimicrobianos (PGA).

**Método:**

Este estudio prospectivo, realizado entre octubre de 2023 y octubre de 2024, utilizó datos de la Red Brasileña de Enfermeras para la Lucha contra la Resistencia Antimicrobiana (REBRAN) en *Instagram* ^®^. La interacción se midió quincenalmente mediante métricas de la plataforma como seguidores, impresiones, visualizaciones, alcance e interacciones. La correlación entre los miembros de REBRAN, los seguidores y el contenido con mayor interacción se analizó mediante estadística descriptiva y el coeficiente de correlación de Pearson.

**Resultados:**

El número de miembros aumentó de 214 a 436 (136%) y el de seguidores de 507 a 1.500, con una fuerte correlación positiva (r = 0,97). El contenido lúdico, como las analogías con películas y fechas conmemorativas, tuvo mayor repercusión que el contenido técnico. El mejor rendimiento se registró en junio de 2024, con 15.000 impresiones, 209 visualizaciones y un alcance de 7.400 cuentas.

**Conclusión:**

Los contenidos lúdicos e interactivos en las redes sociales favorecen la participación de los usuarios en torno a la RAM, ampliando la concienciación e incentivando prácticas basadas en la evidencia en los PGAs.

## INTRODUCCIÓN

La resistencia a los antimicrobianos (RAM) en humanos y animales representa una de las diez principales amenazas globales en salud^(1)^. El uso racional de los antimicrobianos, liderado especialmente por Programas de optimización del uso de antibióticos (PROA), se reconoce como esencial para prevenir el desarrollo de organismos resistentes^(1,2,3)^.

Los PROA han sido tradicionalmente dirigidos por infectólogos y farmacéuticos. Sin embargo, las organizaciones profesionales y los especialistas en el área recomiendan un enfoque colaborativo e interdisciplinario, con la inclusión de enfermeros^(4,5,6)^, considerando que estos profesionales realizan diversas actividades pertinentes para combatir la RAM en las instituciones de salud^(4,7,8,9,10)^.

A pesar de las acciones de los enfermeros relacionadas con los PROA, sigue existiendo una notable falta de conocimiento y concienciación de estos profesionales brasileños sobre la gestión de antimicrobianos^(11,12)^.

Con el objetivo de acercar, apoyar y fortalecer a los enfermeros para que actúen e influyan en los PROA, impactando positivamente en el control de la RAM, se creó la Red Brasileña de Enfermeros para la Lucha contra la Resistencia a los Antimicrobianos (REBRAN). Se trata de un grupo de cooperación técnica formado, principalmente, por enfermeros, con el objetivo de fomentar debates científicos, desarrollar investigaciones y contribuir a la difusión del conocimiento y al compromiso de los enfermeros ante la problemática de la RAM en Brasil. Los enfermeros interesados en el tema pueden afiliarse a REBRAN. Este grupo de cooperación cuenta, entre sus canales de comunicación, con una cuenta de tipo “profesional” en la red social *Instagram* ^®^ para la difusión de contenidos relacionados con la RAM y el PROA^(13)^.

Las redes sociales se han consolidado como una herramienta de comunicación de gran alcance, que permite la rápida difusión de información, la interacción con públicos específicos y la participación de los profesionales de la salud^(14,15)^. Plataformas como *Instagram* ^®^ permiten no solo compartir contenido, sino también crear experiencias interactivas y lúdicas, que pueden favorecer la comunicación, contribuyendo a fortalecer la concientización sobre temas como la RAM^(16,17)^.

El desempeño de una red social puede medirse mediante métricas informativas, como la visibilidad (o visualizaciones), la influencia, la participación y el compromiso^(15)^. Las *visualizaciones* se definen como indicadores de la frecuencia con la que se muestran los contenidos^(18)^. La *influencia* es la capacidad de impactar en actitudes y comportamientos^(19)^. Por su parte, la *participación* se refiere al acto de comentar, compartir y responder de manera activa^(20)^. Por último, el *compromiso* del usuario se produce cuando este realiza una acción activa en relación con el contenido de una red social, como hacer clic, comentar o dar “me gusta” —acciones conocidas como “interacciones”. Estas interacciones se miden en relación con el alcance de la publicación o el número de seguidores, lo que da como resultado la llamada “tasa de compromiso”^(21)^. En general, este indicador se calcula a partir de la suma de la tríada de las “3 C”: “me gusta” (curtir, en portugués), comentar y compartir^(15)^.

Históricamente, el uso de las redes sociales en el ámbito de la investigación en salud fue recibido con cierto escepticismo por parte de la comunidad científica. Sin embargo, en la actualidad ha aumentado el número de publicaciones sobre el tema en revistas científicas, teniendo en cuenta el potencial de esta estrategia^(22,23,24)^. Las aplicaciones de las redes sociales en la investigación en salud son amplias y variadas, y ya incluyen estrategias para aumentar la concientización, incluso sobre la RAM^(25)^. Esta transformación en la comunicación global tiene implicaciones significativas para la salud pública y para la enfermería, ya que las redes sociales pueden utilizarse para dar mayor visibilidad al personal de enfermería en las prácticas de atención de la salud.

Ante este contexto, el objetivo de este estudio fue evaluar la participación de los usuarios de una red social mediante estrategias educativas dirigidas a la sensibilización de los enfermeros sobre la RAM y la PROA.

## MÉTODO

### Diseño del Estudio

Se trata de un estudio observacional prospectivo que evaluó la participación de los usuarios de una red social mediante la implementación de una estrategia educativa.

### Lugar y Población

El estudio se llevó a cabo en Brasil en el período com­prendido entre octubre de 2023 y octubre de 2024. La población objetivo estuvo compuesta por enfermeros afiliados a REBRAN y seguidores de la red en *Instagram* ^®^: “@rebran2022”. Sin embargo, al tratarse de una red social de comunicación abierta, los indicadores de resultados incluyeron a cualquier persona u organización que interactuara con el perfil de REBRAN en *Instagram* ^®^ durante el período propuesto, no solo a enfermeros.

### Estrategia Educativa por Redes Sociales

La estrategia educativa se implementó en *Instagram* ^®^, la principal red social en uso en Brasil^(26)^. La estrategia para la implementación de la intervención se organizó en tres etapas (Figura 1). Todas las acciones fueron desarrolladas por un equipo de investigadores procedentes de diferentes instituciones académicas y servicios de salud, liderados por el equipo de coordinación de REBRAN.

**Figura 1:** Esquema de la estrategia educativa en redes sociales para involucrar a los enfermeros en los contenidos sobre resistencia a los antimicrobianos y el Programas de optimización del uso de antibióticos (PROA) – São Paulo, SP, Brasil, 2025.

**Fuente:** Autores, 2025.

### Recolección de Datos

Para la recolección de datos relacionados con las métricas de *Instagram* ^®^, se utilizó una plataforma específica para el monitoreo de redes sociales, *V-tracker* ^®^, que emplea herramientas que combinan *Social Listening* e *insights* generados por Inteligencia Artificial. La información complementaria, como los *insights* o los indicadores del panel profesional de *Instagram* ^®^ (visualizaciones, interacciones y total de seguidores), fue extraída directamente del área restringida cada quince días, por miembros designados del grupo de trabajo de redes sociales de REBRAN (L.M.A.; C.M.M.T. y T.M.L.S.), entre octubre de 2023 y octubre de 2024.

### Análisis y Tratamiento de los Datos

Tras la recolección, los datos se transfirieron a una hoja de cálculo del programa *Microsoft Excel* ^®^ para su análisis estadístico, en el que se utilizaron métricas específicas de monitoreo de redes sociales (Figura 2).

**Figura 2:** Métricas utilizadas para el monitoreo en redes sociales – São Paulo, SP, Brasil, 2025.

**Fuente:** Autores, 2025.

Se analizó la información en relación con la tendencia temporal a lo largo del período seleccionado (12 meses). Para evaluar la relación entre las variables —el número de seguidores en la página de REBRAN en *Instagram* ^®^ y el número de miembros oficialmente registrados en REBRAN—, se aplicó la correlación de Pearson, que cuantifica la fuerza y la dirección de la asociación lineal entre dos variables cuantitativas continuas^(27)^. El coeficiente de correlación de Pearson (r) varía entre -1 y 1, donde los valores cercanos a 1 indican una fuerte correlación directa (positiva)^(28)^. Por último, se buscó identificar los temas publicados que generaron mayor interacción entre los usuarios de la página de REBRAN en *Instagram* ^®^.

### Aspectos Éticos

Esta investigación fue aprobada por el Comité de Ética en Investigación de la Escuela de Enfermería de la Universidad de São Paulo, mediante el Dictamen n.º 5.953.109 de 2024.

## RESULTADOS

Al inicio de la implementación de la estrategia educativa a través de las redes sociales, REBRAN contaba con 214 miembros registrados oficialmente, cifra que aumentó a 436 al final del período, lo que representa un crecimiento del 136 %. Se observó una tendencia similar en el perfil de *Instagram* ^®^, cuyo número de seguidores pasó de 507 a 1.500, con un incremento continuo a lo largo del período evaluado. El análisis de correlación entre miembros y seguidores reveló una correlación muy fuerte (r = 0,98), lo que demuestra el potencial de la red social como herramienta para ampliar el alcance de la red (Figura 3).

**Figura 3:** Análisis de correlación entre el número de miembros afiliados y el número de seguidores en *Instagram* ^®^ de la Red Brasileña de Enfermeros para la Lucha contra la Resistencia Antimicrobiana – São Paulo, SP, Brasil, 2025.

Los principales temas de interés se publicaron en forma de *post* utilizando la estrategia de *trends* (término que significa “tendencia” y da nombre a los contenidos que alcanzan un pico de popularidad en las redes sociales durante un tiempo determinado). Los temas abordados incluyeron la estructura de REBRAN, la RAM, las Infecciones Asociadas a la Atención de la Salud (IAAS), los antimicrobianos, el papel del enfermero en los PROAs, los hemocultivos, la tendencia de la película “Divertida Mente 2”^(29)^, que relaciona las emociones con el control de la RAM, la tendencia de *Halloween*, que presenta una analogía sobre la RAM, los mitos y verdades sobre la RAM, y la alerta de la Agencia Nacional de Vigilancia Sanitaria (Anvisa) sobre el *Staphylococcus aureus* resistente a la vancomicina.

En cuanto a las métricas de la red social evaluadas a lo largo del período, se observó un crecimiento significativo: las publicaciones, que en octubre de 2023 registraban un promedio de 1600 impresiones, 52,2 visualizaciones y un alcance de 701 cuentas, pasaron en octubre de 2024 a 5400 impresiones, 80,7 visualizaciones y un alcance de 3100 cuentas.

Entre los contenidos publicados, el momento más destacado se produjo en junio de 2024, cuando la estrategia de *posts*que relacionaban ideas y personajes de la película de animación “Divertida Mente 2” (producida por los estudios *Walt Disney, Pictures, Pixar Animation Studios*) con el control de la RAM alcanzó los mejores indicadores de interacción, con un mayor número de impresiones, visualizaciones y alcance (15 000, 209 y 7400, respectivamente). En noviembre de 2023, las acciones de REBRAN para la Semana Mundial de Concientización sobre la Resistencia a los Antimicrobianos, que incluyeron *transmisiones en vivo*, debates y materiales educativos, también presentaron resultados relevantes (4.800 impresiones, 161 visualizaciones y un alcance de 1.700 cuentas). Ya en octubre de 2024, las publicaciones relacionadas con *Halloween*, con un enfoque lúdico sobre la RAM, obtuvieron un desempeño significativo en alcance y participación (4.600 impresiones, 54 visualizaciones y un alcance de 3.300 cuentas).

Por el contrario, el rendimiento más bajo en términos de interacciones totales y alcance se observó en enero de 2024, cuando se publicó sobre las IAAS, específicamente sobre la cadena epidemiológica (impresiones, visualizaciones y alcance).

## DISCUSIÓN

Las redes sociales se consideran una de las principales herramientas para generar participación en la sociedad actual. Sin embargo, no se aprovechan en todo su potencial. Un estudio de revisión sistemática publicado en 2022 investigó la eficacia de las intervenciones para mejorar la concienciación del público sobre la RAM, así como qué intervenciones influyen más en el comportamiento del público. Esta revisión no encontró, entre los estudios elegibles, ninguno que hubiera utilizado recursos de las redes sociales en sus estrategias de intervención^(30)^.

El uso de las redes sociales para sensibilizar, involucrar e informar a los profesionales e incluso a los usuarios de los sistemas de salud es una herramienta poderosa que podría convertirse en un pilar en la lucha mundial contra la RAM^(31)^, dado que tiene un gran alcance, bajo costo y alta capacidad de difusión. Esta estrategia puede ser particularmente importante en países de ingresos bajos y medios, donde el acceso a la alfabetización en salud no siempre llega a toda la población^(31)^.

La transformación que han desencadenado las redes sociales permite una comunicación rápida en salud, con el desarrollo de herramientas estratégicas y dirigidas a públicos específicos con gran alcance y bajo costo. Sin embargo, es necesario encontrar un equilibrio entre los beneficios de estas estrategias y los daños potenciales relacionados con la desinformación en línea^(32)^.

Las redes sociales son, actualmente, un espacio ambivalente en lo que respecta al intercambio de información para los profesionales de la salud. Si, por un lado, ayudan a la rápida difusión de información más allá de las fronteras geográficas, influyen en las actitudes y aumentan la concientización de los usuarios, por otro, pueden utilizarse para la propagación de noticias falsas y desinformación^(33)^. Una revisión señaló que las noticias falsas y la desinformación tienen más probabilidades de difundirse en las redes sociales en comparación con las publicaciones de divulgación científica basadas en la evidencia. Además, estas noticias falsas pueden favorecer la asimilación de concepciones inexactas, lo que compromete la comprensión futura, especialmente cuando se comparan con contenidos precisos difundidos por perfiles educativos^(34)^.

Teniendo esto en cuenta, es necesario que los profesionales de la salud se apropien de los mecanismos de alcance de las redes sociales para difundir información con base científica y promover las mejores prácticas basadas en la evidencia en los servicios de salud. Al respetar los límites éticos y legales que rigen la práctica profesional, es posible lograr reconocimiento social y profesional, demostrando compromiso con la divulgación científica y su aplicación en la atención de la salud^(35)^. Por lo tanto, el uso deliberado y planificado de las redes sociales para aumentar la información correcta en materia de salud, incluyendo información para profesionales, como en el caso del presente estudio, debe considerarse como parte de amplias estrategias de concientización sobre la RAM.

En este estudio, se observó que las publicaciones de carácter lúdico y divertido dieron lugar a mejores métricas de participación, lo que sugiere que esta puede ser una vía prometedora para futuras publicaciones destinadas a la sensibilización sobre la RAM. Esta observación se ve corroborada por experiencias internacionales, como el proyecto español SWICEU (grupo de más de 30 universidades de España y Portugal, patrocinado por la Sociedad Española de Microbiología), que utilizó infografías, videos y gamificación inspirados en la cultura pop para sensibilizar a los jóvenes sobre la RAM, con resultados significativos en cuanto a participación y alcance^(16)^.

Otro aspecto relevante es el papel de las redes sociales en la interacción entre profesionales. Los estudios señalan que plataformas como *Twitter* ^®^ (ahora X^®^) y *Facebook* ^®^ amplían la comunicación científica en tiempo real, estimulando el intercambio de experiencias y la consolidación de buenas prácticas en el control de infecciones y el uso de antimicrobianos. En un estudio realizado por Pisano et al.^(36)^, se observó que los contenidos educativos en las redes sociales no solo aumentaron el conocimiento de los residentes médicos sobre los antimicrobianos, sino que también estimularon el uso de recursos clínicos basados en protocolos, lo que destaca el potencial pedagógico de estas herramientas.

Además, cabe destacar que el perfil de los miembros de REBRAN, compuesto en su mayoría por enfermeros que trabajan directamente en la prevención y el control de las IAAS, puede haber influido en el menor número de accesos a las publicaciones que abordaban aspectos teóricos de algunos temas. Una de las hipótesis sobre este resultado es que estos enfermeros consideren que ya tienen un dominio completo de estos temas o que la forma en que se presentaron no se consideró lo suficientemente innovadora o atractiva como para generar interacción. Así, los contenidos más creativos y accesibles pueden tener un mayor impacto, especialmente en el fortalecimiento del compromiso con la temática^(14)^.

Por último, cabe destacar que *Instagram* ^®^, al ser una red social de carácter más informal y dinámico, favorece el intercambio de experiencias, fomenta un proceso de enseñanza colaborativa y ofrece diversos recursos para el desarrollo de contenidos^(37)^. Esta característica amplía su potencial como herramienta de enseñanza-aprendizaje y de concientización sobre RAM, lo que puede reforzar su papel estratégico para los profesionales de la salud y para la población en general.

Este estudio presenta limitaciones, como la dificultad de identificar entre los miembros de la REBRAN a aquellos que fueron más interactivos en la red social, lo que no permite detallar las características de los perfiles en relación con la participación en las diferentes actividades. Debido a que la red social es abierta, hubo la limitación de no poder reconocer si las interacciones fueron realizadas exclusivamente por enfermeros. Por último, el período del estudio se restringió a un año, lo que no permite extrapolar conclusiones sobre la sostenibilidad a largo plazo de esta estrategia de sensibilización.

## CONCLUSIÓN

Los hallazgos de este estudio demuestran que las redes sociales, en especial *Instagram* ^®^, pueden constituir herramientas estratégicas para promover la concientización sobre la RAM. El análisis de las métricas reveló que las publicaciones lúdicas logran una mayor participación, lo que sugiere que la adopción de lenguajes creativos puede potenciar el impacto de las campañas digitales en salud.

En este sentido, iniciativas como la desarrollada por REBRAN demuestran que la comunicación científica puede ser innovadora, atractiva y efectiva, representando un modelo que puede expandirse y replicarse en otros contextos de salud pública.

## DISPONIBILIDAD DE DATOS

El conjunto de datos que respaldan las conclusiones de este estudio está disponible previa solicitud al autor correspondiente.
